# Beyond the Surface: Interconnection of Viscosity, Crystal Growth, and Diffusion in Ge_25_Se_75_ Glass-Former

**DOI:** 10.1021/acs.jpcb.4c04268

**Published:** 2024-10-07

**Authors:** Jaroslav Barták, David Vaculík, Michaela Vceláková, Simona Martinková, Torsten Wieduwilt, Markus A. Schmidt, Michal Kurka, Stanislav Slang, Karel Palka, Petr Koštál, Petr Belina, Pavla Honcová, Jirí Málek

**Affiliations:** † Department of Physical Chemistry, University of Pardubice, Studentska 573, 53210 Pardubice, Czech Republic; ‡ Department of Inorganic Technology, University of Pardubice, Doubravice 41, 53210 Pardubice, Czech Republic; § Leibniz Institute of Photonic Technology, Albert-Einstein-Str. 9, 07745 Jena, Germany; ∥ Abbe Center of Photonics and Faculty of Physics, Friedrich-Schiller-University Jena, Max-Wien-Platz 1, 07743 Jena, Germany; ⊥ Otto Schott Institute of Material Research, Friedrich-Schiller-University Jena, Fraunhoferstr. 6, 07743 Jena, Germany; # Center of Materials and Nanotechnologies—CEMNAT, University of Pardubice, Nam. Cs. Legii 565, 532 10 Pardubice, Czech Republic; ∇ Department of General and Inorganic Chemistry, Faculty of Chemical Technology, University of Pardubice, Studentska 573, 532 10 Pardubice, Czech Republic

## Abstract

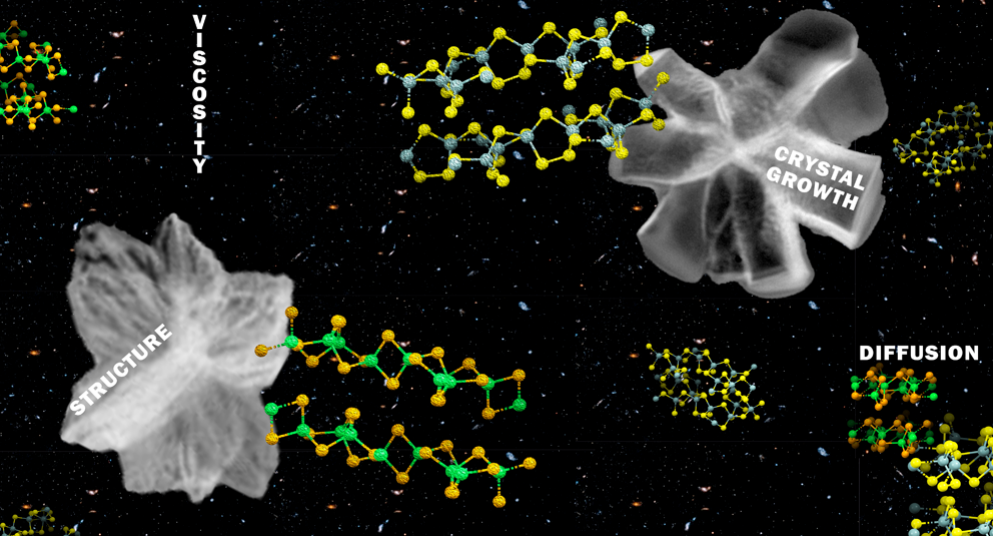

The knowledge of viscosity behavior, crystal growth phenomenon, and diffusion is important in producing, processing, and practical applications of amorphous solids prepared in different forms (bulk glasses and thin films). This work uses microscopy to study volume crystal growth in Ge_25_Se_75_ bulk glasses and thermally evaporated thin films. The collected growth data measured over a wide temperature range show a significant increase in crystal growth rates in thin films. The crystal growth is analyzed using near-surface viscosities obtained in bulks and thin films using nanoindentation and melt viscosities measured by a pressure-assisted melt filling technique. The crystal growth analysis provides information on the size of the structural units incorporated into the growing crystals, essential for estimating the diffusion coefficients and explaining the difference in crystal growth rates in bulk and thin films. The crystal growth analysis also reveals the decoupling between diffusion and viscous flow described by the Stokes–Einstein–Eyring relation. Moreover, to the authors’ best knowledge, the manuscript provides the first evaluation estimation of the effective self-diffusion coefficient directly from growth data in chalcogenide glass-formers. The present data show a similar relation between diffusion coefficients (*D*) and crystal growth rates (*u*): *u* ≈ *D*^0.87^, which is found in several molecular glasses.

## Introduction

Chalcogenide glasses have been intensively studied for several decades because of their diverse properties and utilization in various practical applications. These materials, in the form of bulk glasses and thin films, exhibit high transparency and nonlinearities in a broad infrared (IR) region (0.5–18 μm), making them promising materials for IR optics.^1^ They show pronounced optical and electronic transport property changes during switching between amorphous and crystalline states. Therefore, they find the use in data storage devices,^2^ optical switches,^3^ tunable emitters and absorbers,^4^ nonvolatile photonics,^5^ etc. Essential information for the application of glass-forming materials in practice is derived from studies of physical properties, (such as viscosity, diffusion, thermal properties, etc.) and kinetic phenomena (structural relaxation, crystallization) taking place in these materials.

In this study, we focus on the Ge–Se system. Recently, the Ge–Se system has been intensively investigated, mainly in terms of optical and optoelectronic properties. One possible application is solar photocells.^6^ It can also be used in water electrolysis as part of a photocathode.^7^ This system also has the potential to be anode in sodium-ion and potassium-ion batteries.^8^ In general, glasses of this system are used for optical and holographic devices to record information,^9^ optical elements for infrared (IR) optics, waveguides,^10^ photodetectors,^11^ glass fiber detectors for CO_2_,^12^ radiation dosimeters,^13^ phase transformation temperature-sensing devices,^14^ materials useful for inkjet printing for temperature-sensing sensors,^15^ or as a material suitable for Ovonic threshold switching.^16^ The Ge_25_Se_75_ composition that is investigated in this article has been studied for possible utilization in optical filters^17^ or in resistance random access memory applications.^18^

It is evident that for different applications of these materials, the samples need to be prepared in various forms (bulks, fibers, thin films) and different states (amorphous vs crystalline). Therefore, studies of the physical properties and kinetic phenomena in glass-forming materials must be performed on various types of samples and compositions. Such information is then necessary for further utilization of these materials in practical applications such as phase change materials for data storage where the amorphous-to-crystalline transformation is essential or, on the contrary, for utilization of these materials in optics where a stable glass needs to be obtained and the crystallization process must be prevented.

The present study shows differences in crystal growth rates in bulk samples and thin films. In some chalcogenide glass-formers,^19−21^ surface crystals grow faster than those formed in the volume of the sample, and the surface crystal growth rates are similar to growth rates measured in thin films. This phenomenon is attributed to the higher mobility of the structural units near or at the free surface. This behavior is typical for organic molecular glass-formers,^22,23^ but it was also reported for metallic^24^ and oxide^25^ glasses. Nevertheless, a higher surface mobility cannot be expected in general. Some studies on oxide glasses^26^ provide comparable growth rates (within the experimental errors) for surface and volume crystals. In chalcogenide systems, we can find some works^27−29^ revealing even lower growth rates of surface crystals in comparison to volume crystals. Such behavior was also reported for chalcogenide thin films.^30^ It is clear then that there is no general rule to expect faster/slower surface or near-surface mobility in different glass-forming systems. Therefore, careful analyses of various systems are still needed to better understand the growth vs mobility relation.

**Figure 1:**
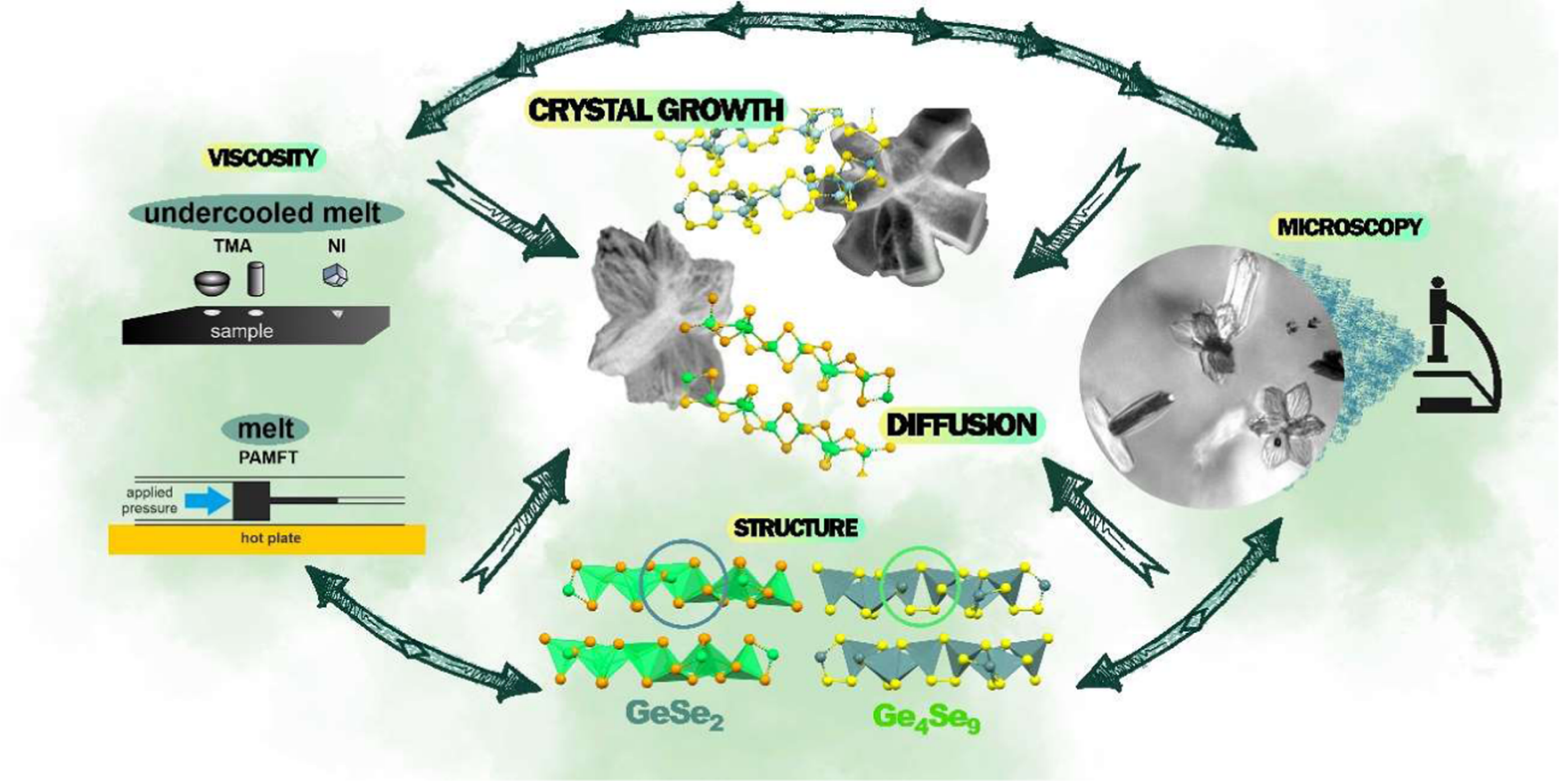
Interconnection of direct observation of crystal growth, viscosity measurements, and structure study leading to essential information on crystal growth phenomenon and diffusion in Ge_25_Se_75_ amorphous material.

Our study interconnects the information about the structure, crystal growth, and viscous flow to provide detailed knowledge about the crystal growth phenomenon and diffusion in the Ge_25_Se_75_ amorphous material, as illustrated in Figure 1. We focus on viscosities and direct crystal growth in bulk samples and thermally evaporated thin films. Viscosities were measured using nanoindentation on the amorphous samples’ surface in the region of the undercooled melt and pressure-assisted melt filling technique in the melt region. The data complement the previously published data in bulk samples in the undercooled melt region.^31,32^ Crystal growth data were obtained using infrared microscopy measurements, showing a change in crystal morphology within the broad studied temperature region (250–560 °C). Moreover, the crystal growth analysis allowed us to obtain the self-diffusion coefficients for the structural units incorporated into the growing crystals. To the best of our knowledge, this information is shown for the first time in the case of chalcogenide glasses and thin films. Diffusion and crystal growth analyses revealed similarities in the crystal growth rates and diffusion coefficients found in molecular systems.

## Experimental Section

Chalcogenide Ge_25_Se_75_ bulk glass was prepared by using the conventional melt-quench method. An appropriate amount of pure elements (5N, HiChem, Czech Republic) was weighed into a clean silica ampule. The ampule was evacuated (2 × 10^–3^ Pa), sealed, and placed in a rocking furnace at 950 °C for 20 h. Subsequently, the temperature was lowered to 800 °C for 2 h, and the ampule was removed from the furnace and cooled down in iced water to obtain bulk glass. The bulk material was used for crystal growth study, as well as for thin film preparation.

Thin films were prepared using the thermal evaporation technique (model UP-858, Tesla Corp.) on microscopy slides (soda-lime substrates). The substrates were fastened and rotated (employing a planetary rotation system) in an evacuated (2 × 10^–3^ Pa) chamber. The films were deposited at a rate of 1–2 nm s^–1^, which was measured by the quartz crystal microbalance technique (STM-2 Inficon). The final thickness of the thermally evaporated films was 1000 and 210 nm.

The composition of the prepared bulk glass, thin films, wires for PAMFT measurements, and samples after PAMFT measurements was verified using an energy-dispersive X-ray (EDS) microanalyzer (Aztec X-Mac 20, Oxford Instruments, 5 kV for TF and 20 kV for bulks) coupled with a scanning electron microscope (SEM; LYRA 3, Tescan). The results from EDS analysis are shown in Supporting Information in Table S5.

X-ray diffraction (Rigaku MiniFlex 600 with Bragg–Brentano θ–2θ geometry; Cu Kα λ = 1.5418 Å, *U* = 400 kV, and *I* = 15 mA) was used to confirm the amorphous nature of the prepared bulk glasses and thin films. The same XRD was also used to characterize the crystalline phases grown in the samples. The XRD scans were collected using the ultrafast detector Dtex ultra in the range 2θ = 5–65° with a scanning rate of 10° min^–1^, and step of 0.02°.

Viscosities in bulk samples and thin films were studied in a wide temperature range using two different techniques: pressure-assisted melt filling technique (PAMFT) and nanoindentation (NI). PAMFT was used to study viscosities in the melt region. A prepared chalcogenide wire (60–80 μm in diameter, 1–2 mm in length) was placed into a silicate glass capillary with an inner diameter of 80 μm and an outer diameter of 200 μm. The capillary was then spliced with a filling capillary of an inner diameter of 5.8 or 10 μm. The capillaries were then connected to the pressure system and purged for 30 min with pure Ar(5.0) to remove air from the capillaries. The capillaries were then placed on a horizontal hot plate (with temperature stability ±1 °C), and Ar pressure (5 and 40 bar) was applied to the free end of the capillary to push the chalcogenide melt into the filling capillary. After the predefined time interval, the capillary was removed from the hot plate and cooled and the length of the filled material in the small capillary was measured. The procedure was repeated several times to obtain filling length/filling time data used for viscosity calculations. More details about the method can be found elsewhere.^33,34^ The NI technique was used to measure near-surface viscosity data in thin films and bulk samples in undercooled melt and glass regions. NI system Hysitron TI Premier (Bruker co.) equipped with xSol 600 heating stage (temperature stability ±0.2 °C) was used for the measurements. The samples were purged by N_2_ and heated in the heating stage at 20 °C min^–1^ to reach the set temperature. Standard sapphire Berkovich indenter (three-side pyramid with tip angle Ψ = 142.3°) was used for the NI experiments.

The thermal behavior of prepared bulk material and thin films was studied by using a differential scanning calorimeter (DSC, Sensys Evo 3D, Setaram Co.). Samples were measured in opened silica glass ampules at a heating rate of 10 °C min^–1^.

The crystal growth in bulk samples and thin films was monitored under isothermal conditions using an Olympus BX51 optical microscope equipped with an infrared camera XM10. The samples were previously heat-treated in a computer-controlled furnace (temperature stability of ±0.5 °C) at selected temperatures for specified times. After annealing, the samples were quickly cooled and measured under the microscope. The thin film samples were also studied in situ. The samples were annealed in the Linkam THMS600/720 heating stage (temperature stability ±0.1 °C), and the micrographs were directly collected during the annealing.

## Results and Discussion

### Viscosity in Ge_25_Se_75_

**Figure 2:**
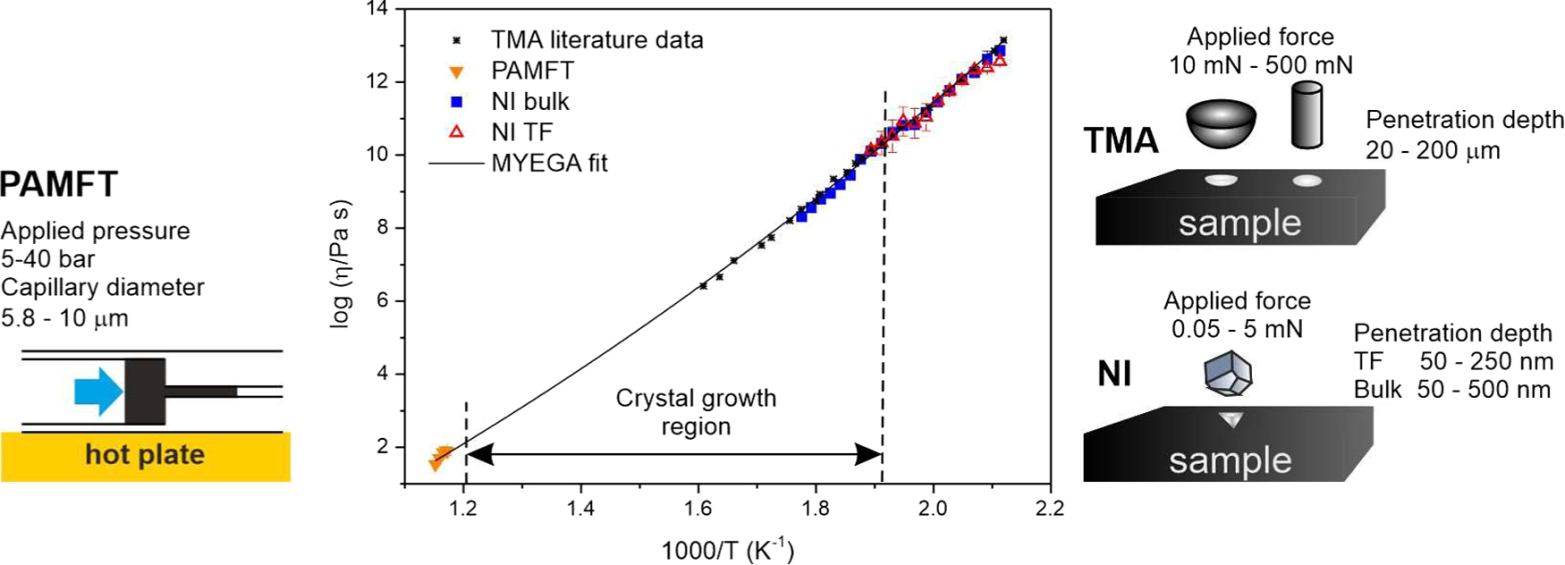
Temperature (*T*) dependence of near-surface viscosity (η) in the undercooled melt region in Ge_25_Se_75_ bulks and thin films (TF, 1000 nm in thickness) obtained by nanoindentation (NI), and melt viscosities obtained by the pressure-assisted melt filling technique (PAMFT, the scheme of the method is depicted). The figure compares literature data in bulks measured by thermomechanical analysis (TMA) and published by Honcová et al.^31^ The solid line represents the MYEGA model calculated according to eq 1. Schematics and differences in settings and measured penetration depths using TMA (used for bulk viscosity measurements only in bulk samples) and NI (used for near-surface viscosity measurements in both bulks and TF) are also shown.

Knowledge of the viscosity behavior in amorphous materials, not only chalcogenides, is essential for processing and describing the kinetic processes taking place in these materials. Researchers can use many experimental techniques to measure viscosity in bulk materials in the region of glass and undercooled melt regions.^35^ Most of the techniques use a penetration method with indenters of different shapes. The penetration depth is measured by thermomechanical analyzers (TMA) and usually reaches 20–200 μm (the scheme is shown in Figure 2).^36^ Due to classical indenters’ size and penetration depths, these techniques cannot be applied to study the viscosity behavior in thin films. The literature provides only a few works focusing on viscosity measurements in chalcogenide thin films.^20,37^

We focus on the measurements of near-surface viscosity in Ge_25_Se_75_ amorphous materials prepared in different forms. The near-surface viscosities were measured in thin films (TF) of 1000 nm thickness and bulk samples using nanoindentation (NI). The NI system offers a unique tool for studying the thin film viscosity behavior. The near-surface viscosities were measured using Berkovich indenter by NI (the scheme is shown in Figure 2), where the viscosity value was obtained from the dependence of penetration depth on penetration time (the Supporting Information provides more details on the NI measurements). We obtained near-surface viscosity data in the range of 10^8^–10^13^ Pa·s (*T* = 200–290 °C) in bulk samples and 10^10^–10^13^ Pa·s (*T* = 200–255 °C) in TF. In general, the thickness of the TF samples limited the viscosity measurements; hence, we used only the TF of the 1000 nm thickness. The penetration depth increased with an increasing temperature (decreasing viscosity), and the TF substrate influenced the measurements. Therefore, the viscosity region in TF is narrower than that in bulk samples. Table S2 in the Supporting Information summarizes all of the measured near-surface viscosity data in bulk samples and TF. The obtained near-surface viscosities correspond well to the viscosity data found for the undercooled melt and glass region in bulk samples.^31,32^Figure 2 compares the near-surface viscosities studied by NI with the bulk viscosities measured by thermomechanical analysis (TMA), previously published by Honcová et al.^31^ All of the data correspond well within the experimental errors. Such a comparison reveals that the near-surface viscosity data in bulks and TF agreed well with the viscosities in bulks.

We also measured melt viscosities to complete the viscosity data in the Ge_25_Se_75_ glass-former. We studied the melt viscosities using the pressure-assisted melt filling technique (PAMFT).^33,34^ PAMFT uses the filling of molten material into thin capillaries with small cores (typically several micrometers; the scheme is shown in Figure 2) under high pressure (see the Supporting Information for more details). We obtained the viscosity values in Ge_25_Se_75_ melts in the 10^1.5^–10^1.9^ Pa·s range (Table S2 in the Supporting Information). Figure 2 shows all of the viscosity data in the Ge_25_Se_75_ materials.

We used the Mauro-Yue-Ellison-Gupta-Allan (MYEGA) equation^38^ to fit the viscosity data in the Ge_25_Se_75_ system:
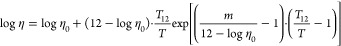
1where η_0_ is the viscosity at infinite temperature, *m* is the steepness index, and *T*_12_ is the temperature where the viscosity reaches the value of 10^12^ Pa·s, also known as viscosity glass transition temperature. Because the viscosity data of Ge_25_Se_75_ bulks and TF corresponded well in the undercooled melt region, we fit all of the data together with one model covering the glass, undercooled melt, and melt regions (full line in Figure 2). We obtained the MYEGA parameters: log (η_0_/Pa·s) = −7.6 ± 0.4; *m* = 27.7 ± 0.4; *T*_12_ = 488.8 ± 0.4 K. The low value of the steepness index means that Ge_25_Se_75_ is a relatively strong glass-former, while most chalcogenide materials show higher values of that parameter.^35^ This means that Ge_25_Se_75_ viscosity in the undercooled melt region is less sensitive to temperature changes than is typical for other chalcogenide materials. The strong behavior is usually attributed to materials with rigid three-dimensional structures.^39^ Hence, the structure of the studied material is probably more interconnected than the structure of most other chalcogenides. We showed previously^35^ that the temperature dependence of viscosity for stronger chalcogenide glass-formers is represented better by the MYEGA equation^38^ rather than the classical Vogel–Fulcher–Tammann (VFT) equation.^40^ This is connected with the shape of curves representing the mentioned equations and with the expected better uniformity of viscosity behavior in the melt region than in the undercooled melt region for chalcogenides.^34^ In the case of Ge_25_Se_75_, the VFT, and MYEGA fits are comparable within the errors of their parameters (see Supporting Information Figure S2 and Table S3). The VFT showed a slightly worse coefficient of determination.

### Crystal Growth in Ge_25_Se_75_ Amorphous Samples

We followed the crystal growth in Ge_25_Se_75_ samples in different sample forms: bulk samples and thin films of 210 and 1000 nm thickness. The following sections focus on crystal growth morphology, changes in the crystalline structure, and the description and prediction of crystal growth in a wide temperature range.

#### Crystal Growth Morphology

We followed the crystal growth in Ge_25_Se_75_ bulk samples and evaporated thin films with two different thicknesses (210 and 1000 nm) by using infrared microscopy (IR-M). The crystals formed in bulks grew in the volume of the samples; we found no crystals at the samples’ surface. The crystals in TF were formed somewhere under the free surface and were limited by the film thickness in the normal direction. Therefore, they grew mainly in the lateral direction and did not exceed the TF’s thickness. We checked this phenomenon using SEM. The partially crystallized TF samples showed that the crystals were still covered by a thin amorphous layer, as shown in Figure S4 in the Supporting Information. When we detached the TF from the substrate and examined it from the “bottom” (means from the side that was in contact with the substrate), the crystal structure was clearly visible. This supports the information that crystals in TF were formed beneath the free surface. Therefore, the crystal growth in TF also corresponds to the so-called volume crystal growth similar to bulk samples, even if the volume in TF is very small due to the film thickness.

**Figure 3:**
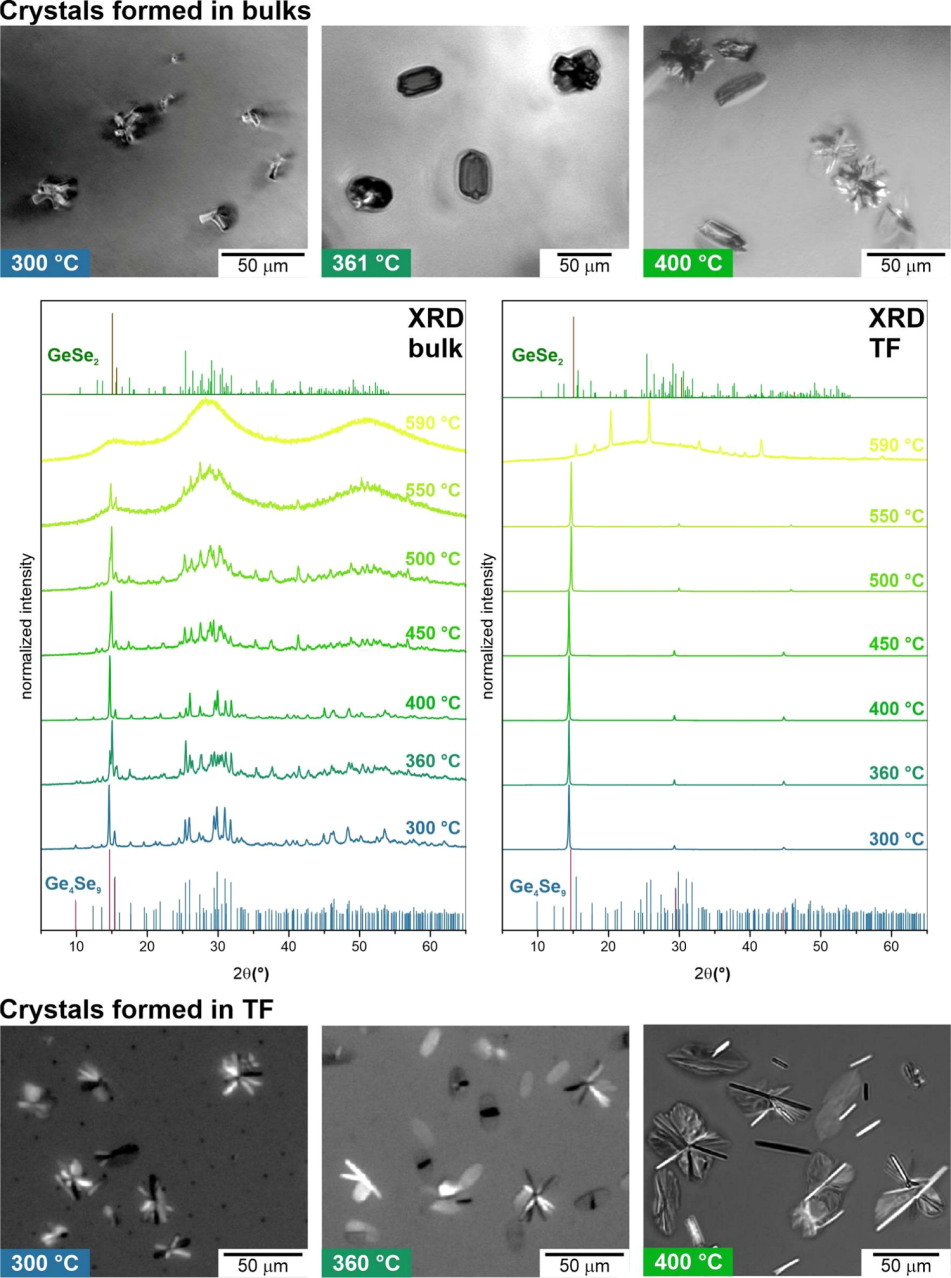
XRD diffractograms of crystalline phases formed in bulk samples and thin films annealed at different temperatures. The XRD patterns were then taken at room temperature. The diffraction patterns are compared with data from the ICDD PDF-2 sheets of GeSe_2_ (nr. 01–084–4687) and Ge_4_Se_9_ (nr. 01–070–7665). The figure also shows typical crystals grown in Ge_25_Se_75_ bulk samples and thin films captured at different temperatures.

Figure 3 shows typical crystals grown in bulk samples and thin films, respectively. A comparison of crystals formed in TF of different thicknesses is shown in Figure S5 in the Supporting Information. The crystal morphology and structure change with the temperature, as is visible, especially for the crystals formed in the bulk samples (Figure 3). This phenomenon is not surprising in chalcogenide glasses and can also be observed in the analog sulfur system (Ge–S).^29,41^ Regarding the crystal growth in bulk samples, Azoulay^42^ and Stolen^43^ showed that in the Ge–Se system (with Ge content <33 atom %), an intermediate phase, called ϕ-phase, is formed. The ϕ-phase has a composition of approximately 30 atom % of Ge in Se^42,43^ and melts incongruently at around 385 °C.^43^ Later, Fjellvag^44^ described the ϕ-phase structure as Ge_4_Se_9_, forming a corner-shared GeSe_4_-tetrahedra via Se_2_ dimers with short Se–Se bonds. We observed a similar transition in the presented study, as Stolen^43^ and Azoulay^42^ described.

In Ge_25_Se_75_ bulk samples, at low temperatures (below 350 °C), the Ge_4_Se_9_ crystals formed rosette-like structures growing from one central point (Figure 3). With increasing temperature (>350 °C), they grew as single small pellets and rarely formed the rosettes (Figure 3). Here, we have to mention that the structure of Ge_4_Se_9_ is very similar to that of monoclinic GeSe_2_. The small difference between these two phases (Ge_4_Se_9_ and GeSe_2_) is that in GeSe_2_, half of the tetrahedra in the crystalline matrix form dimers by edge-sharing.^44^ The structure of GeSe_4_ tetrahedra sharing within the Ge_4_Se_9_ and GeSe_2_ crystals is shown in Figure 1. In the temperature region 350–380 °C, these tetrahedra units probably transform from the Ge_4_Se_9_ arrangement to that of GeSe_2_. Above 380 °C, crystals of GeSe_2_ started to form. In the case of TF (Figure 3), we observed crystals with a similar morphology in the whole studied temperature range. This phenomenon might be caused by the limited thickness of the thin films. X-ray diffraction (XRD) analysis (Figure 3) showed changes in the structures of the formed crystalline phases with temperature in bulk samples and TF (Figure 3). The measured XRD patterns in Figure 3 are compared with the XRD data from the International Centre for Diffraction Data (ICDD) from Powder Diffraction File (PDF-2) sheets of GeSe_2_ (nr. 01–084–4687) and Ge_4_Se_9_ (nr. 01–070–7665). Due to the similar structures of Ge_4_Se_9_ and GeSe_2_, many diffraction lines overlap. Nevertheless, the most significant change in XRD patterns for bulk samples occurs with the two most intensive peaks in the 2θ = 14–16° range (marked red in Figure 3). A small shift of the peaks at 14.65 and 15.43°, corresponding to the Ge_4_Se_9_-phase, toward 15.05 and 15.55°, corresponding to the GeSe_2_-phase, shows the transformation of the phases. Also, a peak at 9.92° that is characteristic only for Ge_4_Se_9_ (also marked in Figure 3), disappears with increasing temperature above 400 °C. In the case of TF, the XRD patterns are strongly affected by the prior orientation caused by the small thickness of the films. Nevertheless, the shift of the main peak at position 14.65° (Ge_4_Se_9_) toward 15.05° (GeSe_2_) is noticeable. There occurs also a shift of a small peak at 29.46° that corresponds only to the Ge_4_Se_9_ phase to the position of 30.31° corresponding to the GeSe_2_ phase. The XRD experiments (Figure 3) also showed broad and scattered diffraction peaks in XRD patterns above 500 °C in bulk and above 550 °C in TF. The broadening of the XRD diffraction peaks is probably caused by the overlapping GeSe_2_ growth and fusion processes. This is in good agreement with the literature data. Several authors^42,43,45^ provide the melting temperature of Ge_25_Se_75_ material to be in the range of 497–640 °C.

#### Crystal Growth Kinetics

We studied the volume crystal growth in Ge_25_Se_75_ amorphous samples prepared in different forms (thin films of 1000 and 210 nm thickness and bulks). We evaluated the crystal growth rates (u) from the linear time-crystal size dependences in a wide temperature range (250–560 °C). The evaluation of the crystal growth rate is shown in Figure S3 in the Supporting Information, and the crystal growth rates are summarized in Tables S4 and S5.

We applied the simple Arrhenius model for the crystal growth rate (*u* = *u*_0_ exp(−*E*_G_/*RT*), where *u*_0_ is the preexponential factor) to obtain the apparent activation energy of crystal growth *E*_G_, as is shown in the Supporting Information in Figure S6. The slopes of the straight lines (linearized Arrhenius model) in Figure S6 revealed the apparent activation energies of crystal growth *E*_G_ = 107.6 ± 0.8 kJ mol^–1^ for bulk samples (the decreasing part of the crystal growth rate above 528 °C was not used for the linear fit and will be discussed later) and *E*_G_ = 99.7 ± 0.6 kJ mol^–1^ for TF. We have to note that the simple Arrhenius model is suitable only for estimating the apparent activation energy of crystal growth in a relatively short temperature region. Nevertheless, the model does not include the transportation of structural units from the undercooled melt toward growing crystals. Therefore, the Arrhenius model cannot be used to predict or extrapolate the crystal growth rates in a wide temperature region, so more sophisticated growth models and analyses must be performed.

**Figure 4:**
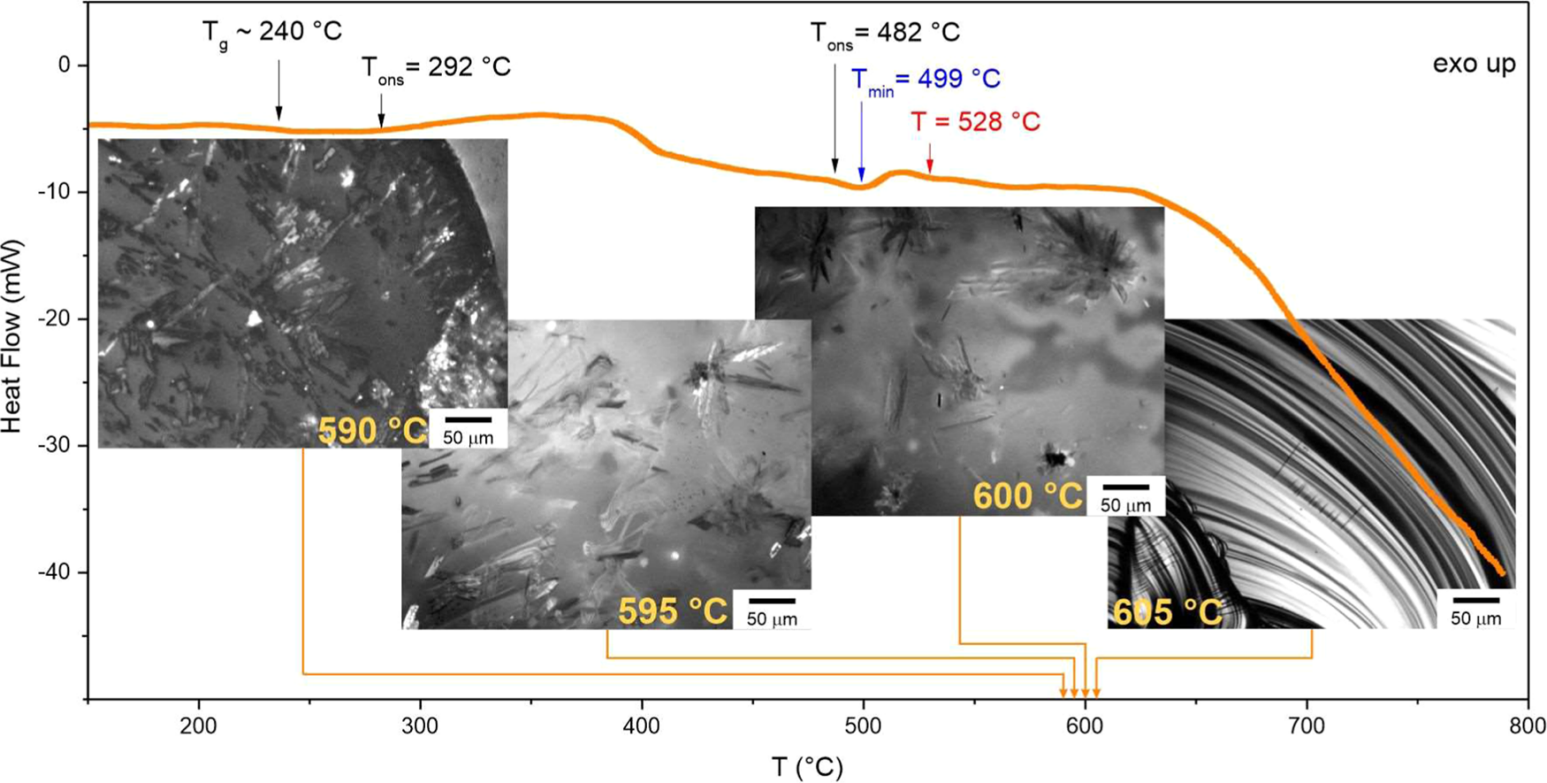
DSC measurement of a bulk sample at a heating rate of 10 °C min^–1^. The photographs represent the evaluation of liquidus temperature *T*_L_ by checking the residual crystals in fully crystalline samples heated to a chosen temperature.

The crystal sizes developed linearly with time (Figure S3 in the Supporting Information). This behavior indicates that the liquid-crystal interface kinetics drives the crystal growth.^46^ We have shown in our previous works^28,47,48^ that the appropriate model can be estimated with knowledge of the temperature dependences of Δ*G* (change in Gibbs free energy between the crystalline and amorphous phase), which can be calculated^49^ from ΔH*_m_ and T*_m_ (enthalpy and temperature of melting of the growing crystalline phase, respectively), and *T*_L_ (liquidus temperature of the material), η (viscosity), and parameters of ξ. The parameter ξ represents a decoupling parameter between viscosity and diffusion coefficient (*D* ≈ η^–ξ^)^50^ representing the transport of structural units from the undercooled melt toward crystal–liquid interphase. As was shown earlier, the crystalline phase changes directly from the ϕ-phase (Ge_4_Se_9_^44^) to GeSe_2_ within a wide temperature range. Due to this phenomenon, we cannot estimate *T*_m_* and Δ*H*_m_* for the ϕ-phase. Therefore, the melting temperature and enthalpy of the crystalline phase were chosen to correspond to the GeSe_2_ phase, ΔH*_m_ = 24 kJ mol^–1^ and T*_m_ = 736 °C.^43^ Regarding the *T*_L_, literature data shows that it varies from 497 to 640 °C according to different authors.^42,43,45^ Therefore, we performed differential scanning calorimetry (DSC) measurements (Figure 4) to characterize the material and find the melting parameters needed for the growth model calculations. The measurements of the as-prepared samples showed a glass transition at *T*_g_ = 240 °C for bulk and similarly at *T*_g_ = 241 °C for TF. The crystallization in these samples is very slow and starts at 292 °C (at a heating rate of 10 °C min^–1^). After the crystallization, there is a small endothermic peak with onset at 482 °C, minimum at 499 °C, and ending at 516 °C. Nevertheless, this melting peak is very low, and crystal growth was observed even above this temperature (up to 559 °C). This means that endothermic melting overlaps with exothermic crystallization, and we cannot determine the melting temperature or the enthalpy of melting or crystallization. Therefore, we had to find the liquidus temperature, *T*_L_ differently. To estimate the *T*_L_, we prepared fully crystalline samples that we annealed at a chosen temperature between 550 and 650 °C for 1 h, and then the sample was rapidly cooled down to room temperature. Subsequently, we checked the sample under a microscope to find the remaining crystalline phase, as indicated in Figure 4. We found the liquidus temperature to be *T*_L_ ∼ 605 °C, which agrees with the findings of Stolen^43^ (*T*_L_ ∼ 607 °C).

**Figure 5:**
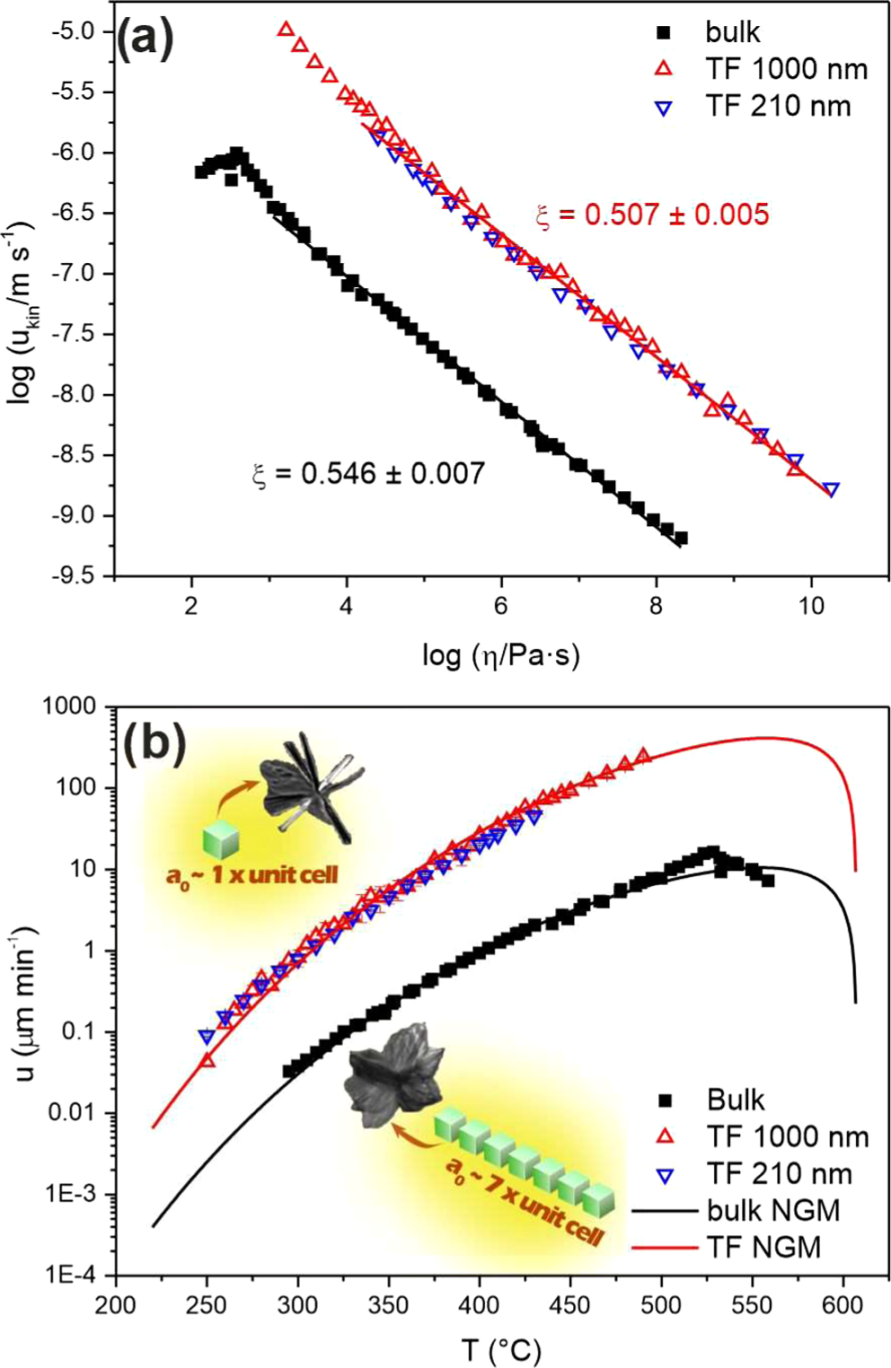
(a) Dependence of log *u*_kin_ on log η in Ge_25_Se_75_ bulk glasses and thin films. (b) Temperature dependence of crystal growth rates in Ge_25_Se_75_ bulk samples and TF described by the NGM.

The decoupling parameter ξ can be estimated from the dependence of the kinetic part (*u*_kin_, eq 2) of the crystal growth rate on the viscosity (η), as is shown in Figure 5a. The *u*_kin_ is defined as:
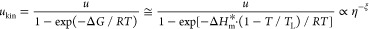
2The change in Gibbs free energy between the crystalline and amorphous phase was substituted using enthalpy of melting of the crystalline phase ΔH*_m_ and the liquidus temperature *T*_L_.^28,49^*R* denotes the universal gas constant (8.314 J mol^–1^ K^–1^).

Figure 5a shows log *u*_kin_ vs log η. The dependence shows a linear course in the viscosity range of 10^10^–10^4^ Pa·s, providing the decoupling parameters: ξ = 0.546 ± 0.007 for bulk samples and ξ = 0.507 ± 0.005 for TF. At lower viscosities (η < 10^4.5^ Pa·s; *T* > 427 °C), the parameters slightly increased to 0.76 ± 0.05 and 0.62 ± 0.02 for bulk samples and TF, respectively. The increase in ξ can be connected to a change in the crystal morphology. The ξ values show a significant deviation from the Stokes–Einstein–Eyring (SEE) relation between diffusion coefficients and viscosity (*D* ≈ η^–1^).^51^ Therefore, parameter ξ needs to be used for further crystal growth analysis.

The temperature dependence of crystal growth rates in Ge_25_Se_75_ can be described by using the normal crystal growth model (NGM). Concerning the decoupling between viscosity and diffusion and the different compositions of the undercooled melt and the growing crystalline phase, the NGM can be expressed as^28,46^
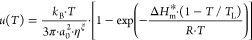
3where *k*_B_ is the Boltzmann constant and *a*_0_ is the only fitting parameter for the model representing the size of structural units incorporated into the crystal during its growth.

Figure 5b shows the fitted NGM to the presented experimental growth data in Ge_25_Se_75_ bulks and TF. We extend the fitted model to a temperature range from *T*_12_ to *T*_m_ as the NGM may be used to predict crystal growth data over a wide temperature range. For the fitting, the lower values of the parameters ξ (0.546 for bulks and 0.507 for TF) were used because they describe the broader range of the data. Using nonlinear fitting, we found the parameters a_0_ of the NGM to be 100.4 ± 0.4 and 14.61 ± 0.10 Å for bulk samples and TF, respectively. According to the *a*_0_ parameters, larger units are incorporated into the crystal–liquid interface of the growing crystals in bulk samples than in TF. These findings contradict the work theory summarized in the work of Slezov,^52^ where the size of the incorporation of structural units is determined only by the composition of the forming crystals. Nevertheless, our results on crystal growth in Ge_25_Se_75_ bulk and thin films propose another explanation. The different sizes of the structural units may originate from the internal stresses in the prepared amorphous samples of bulks and thin films. The adhesion between TF and substrate causes a shear strain that can shorten building units linking up more easily with the crystalline domain, as was described by Stephens^53^ for crystallization in amorphous selenium films. This estimation agrees with the observed crystal growth rates, where bulk growth rates are more than 10 times slower than in TF. The structure of the transported units can be assessed from the cell volumes of the Ge_4_Se_9_ and GeSe_2_ crystals that are similar (*V*_Ge_4_Se_9__ = 1508 Å^3^ from ICDD PDF-2 nr. 01–070–7665; *V*_GeSe_2__ = 1385 Å^3^ from ICDD PDF-2 nr. 01–084–4687). Regarding the parameters *a*_0_ for crystal growth in TF (*a*_0_ = 14.6 Å), a structural unit of a diameter of one to two unit cells diffuses in the TF and incorporates into the growing crystals (Figure 5a). On the other hand, in bulk (*a*_0_ = 100.4 Å), larger aggregates of a diameter of approximately seven to nine units are formed and transported from the undercooled melt to the growing crystals, as illustrated in Figure 5a. Another important finding is the maximum crystal growth rate in bulk samples at 528 °C. Above 528 °C, the growth rates start to decrease rapidly, an unusual phenomenon observed in crystal growth in chalcogenide glass-formers. The phenomenon might be related to the beginning of a slow melting process (Figure 4) that overlaps with growth. According to different authors,^42,43,45^ this explanation can be supported by the literature data on liquidus temperature *T*_L_, which shows a broad interval of 497 to 640 °C.

#### Crystal Growth and Self-Diffusion

The structure of Ge_25_Se_75_ amorphous materials consists of randomly distributed Se-chain and GeSe_4_ tetrahedra units.^54^ As mentioned above, the growing crystals (Ge_4_Se_9_ and GeSe_2_) are built of GeSe_4_ tetrahedra. The structural units (probably formed from the GeSe_4_ tetrahedra) are then transported from the amorphous phase to the growing crystals as a group of Ge and Se atoms. Therefore, an effective diffusion coefficient describes the structural unit transport process and is estimated using the structural unit sizes.

As shown earlier, the kinetic part of the crystal growth rate (corresponding to the diffusion coefficient) strongly decouples from the viscosity. In such a case, diffusion governs the crystal growth and cannot be easily replaced by viscosity. The knowledge of diffusion coefficients in glass-forming materials is rare, especially for chalcogenide glass-formers. The most common relation used in crystal growth theory to describe diffusion is the Stokes–Einstein–Eyring (SEE) relation:^51^
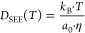
4

The SEE relation describes the diffusion coefficients near the melting temperature well. Nevertheless, as shown in this work and chalcogenide,^20,27,28,47,55^ oxide,^25,56^ and molecular^50,57^ glass-forming systems, the SEE usually breaks down at higher undercooling as well as for surface crystal growth or crystal growth in TF.

**Figure 6:**
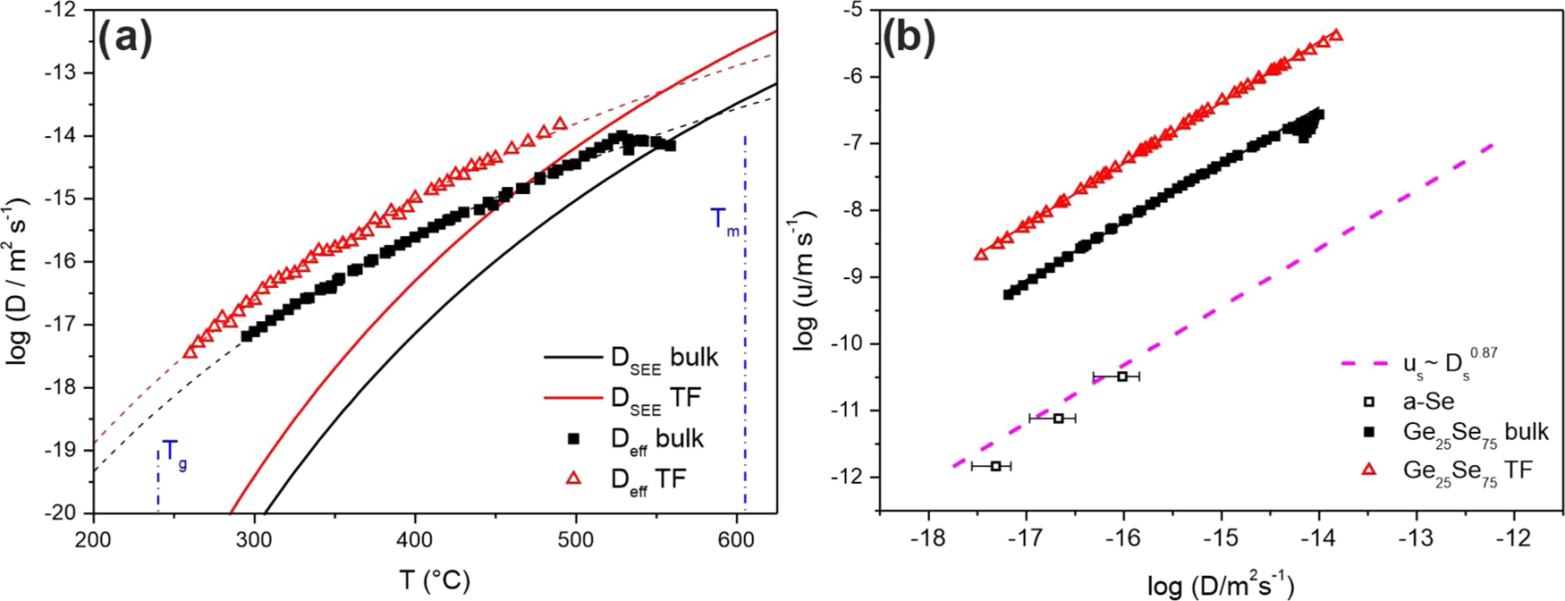
(a) Temperature dependence of calculated diffusion coefficient *D* in Ge_25_Se_75_ bulk samples and thin films. The dashed lines correspond to the Arrhenian fit to the *D*_eff_ data. (b) Volume crystal growth rate as a function of the effective self-diffusion coefficient in Ge_25_Se_75_ bulk samples and TF (present data). Comparison with surface crystal growth rate-surface self-diffusion coefficients relation for amorphous selenium (a-Se)^21^ (hollowed points) and organic glass-formers^22^ (dashed line) is also depicted. Slopes of the linear dependence (solid lines) for the Ge_25_Se_75_ bulks and TF gain values are 0.863 ± 0.007 and 0.912 ± 0.004, respectively.

On the other hand, the effective diffusion coefficients (*D*_eff_) can be independently calculated directly from the experimental data of crystal growth rates:^25,46,51^
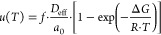
5where *f* reflects the growth mechanism, and for our suggested NGM, *f* = 1. Applying the parameters *a*_0_ found from the NGM, we estimated the effective self-diffusion coefficients (*D*_eff_) in Ge_25_Se_75_ bulk glasses and TF in the region of the undercooled melt (Figure 6a). The higher *D*_eff_ in TF than in bulks was expected since smaller units (according to parameter *a*_0_) were transported from the undercooled melt to the crystal–liquid interface (Figure 5a). The *D*_eff_ is also compared with the diffusion coefficients calculated using the SEE relation (*D*_SEE_; eq 4). The *D*_eff_ is described using a simple Arrhenius equation (Figure 6a) to show the possible trend of D_eff_ on the temperature. This interpretation helps reveal the decoupling temperature where the diffusion deviates from the approximation given by the SEE relation, corresponding to 558 °C for TF and 584 °C for bulks of Ge_25_Se_75_. The decoupling temperatures show that in this Ge_25_Se_75_ particular system, decoupling between diffusion and viscous flow occurs in the whole undercooled melt region starting close to the liquidus temperature *T*_L_ = 605 °C. That differs from the observations in some oxide glasses^25,58^ where the decoupling temperatures reach values of (1.1–1.2)·*T*_g_.

The independently obtained effective diffusion coefficients (*D*_eff_) and their relationship to the measured crystal growth rates can be compared with the experimental data on the surface self-diffusion coefficients found for amorphous selenium^21^ and organic molecular glass-formers.^22^ The effective diffusion coefficients in Ge_25_Se_75_ samples show a corresponding trend as the data on molecular systems (Figure 6b), which is shown for the first time for binary chalcogenide glass-formers. In molecular systems,^21,22^ surface crystal growth rates (*u*_s_) strongly correlated to the surface self-diffusion coefficient (*D*_s_), which can be expressed as *u*_s_ ≈ *D*_s_^0.87^. Figure 6b shows that a similar relation also holds for the volume crystal growth in Ge_25_Se_75_ in TF and startlingly for volume crystal growth in bulk samples, where the exponents gained values of 0.912 ± 0.004 and 0.863 ± 0.007, respectively. This conclusion might be of great interest because the analysis of crystal growth rates (often easier to measure) can provide missing information about the self-diffusion coefficients in chalcogenide glass-formers.

## Conclusions

The analysis of viscosity and volume crystal growth in Ge_25_Se_75_ bulk samples and thin films shown in this work provides important insight into the structure, mobility, and relation between mobility and crystal growth in Ge_25_Se_75_ glass-former. We found that the crystal growth is faster in thin films than in bulk samples of more than 1 order of magnitude, and a significant decoupling of viscosity and kinetic part of the crystal growth rate (corresponding to diffusion) occurs for all of the studied samples within the whole region of undercooled melt. Therefore, we can assume that the diffusion process governs the crystal growth. The crystal growth analysis suggested in this work provides important information about the crystal growth model, describing the data and size of the structural units incorporated into the crystalline phase during the growth. Moreover, the crystal growth analysis allowed us to obtain the effective self-diffusion coefficients for the structural units incorporated into the growing crystals. This evaluation is performed for the first time in chalcogenides and revealed similarities in the relationship between the crystal growth rates and diffusion coefficients found in molecular systems.

## Supporting Information Available

Additional experimental details and methods, and tables with data of measured viscosities and crystal growth rates (PDF)
