# Supplementary material for: Beyond the Surface: Interconnection of Viscosity, Crystal Growth, and Diffusion in Ge25Se75 Glass-Former
Source: J Phys Chem B. 2024 Oct 7;128(41):10286–96. doi: 10.1021/acs.jpcb.4c04268 (PMC11492241; doi:10.1021/acs.jpcb.4c04268)
Supplement: Supplementary file 1 — jp4c04268_si_001.pdf [file jp4c04268_si_001.pdf]

Beyond the Surface: Interconnection of Viscosity, Crystal Growth, and Diffusion in $Ge_{25}Se_{75}$ Glass-Former

Jaroslav Barták[*1], David Vaculík[1], Michaela Vceláková[2], Simona Martinková[1], Torsten Wieduwilt[3], Markus A. Schmidt[3,4,5], Michal Kurka[6], Stanislav Slang[6], Karel Palka[6,7], Petr Koštál[2], Petr Belina[2], Pavla Honcová[2], Jirí Málek[1]*

*corresponding author: jaroslav.bartak@upce.cz

[1]Department of Physical Chemistry, University of Pardubice, Studentska 573, 53210 Pardubice, Czech Republic

[2]Department of Inorganic Technology, University of Pardubice, Doubravice 41, 53210 Pardubice, Czech Republic

[3]Leibniz Institute of Photonic Technology, Albert-Einstein-Str. 9, 07745 Jena, Germany

[4]Abbe Center of Photonics and Faculty of Physics, Friedrich-Schiller-University Jena, Max-Wien-Platz 1, 07743 Jena, Germany

[5]Otto Schott Institute of Material Research, Friedrich-Schiller-University Jena, Fraunhoferstr. 6, 07743 Jena, Germany

[6]Center of Materials and Nanotechnologies - CEMNAT, University of Pardubice, nam. Cs. legii 565, 532 10 Pardubice, Czech Republic

[7]Department of General and Inorganic Chemistry, Faculty of Chemical Technology, University of Pardubice, Studentska 573, 532 10 Pardubice, Czech Republic

Compositional analysis

All prepared samples studied in this work were verified for their composition using energy dispersive X-ray analysis (EDS). We analyzed as-prepared bulk glasses, thin films (TF) prepared by thermal evaporation, and thin wires used for PAMFT measurements to obtain viscosity data in the melt. We also verified the composition of the material filled in thin capillaries after PAMFT measurements to check that there was no phase separation of the studied material during the measurements. The analysis was performed using EDS in several places of the studied samples. The obtained compositions are summarized in Table S1. The standard deviations given in Table S1 show the compositional variation in several tested places in one sample. Nevertheless, the standard deviation in composition determination using the noted EDS detector is about 1 at%.

Table S1 Compositional verification of prepared samples using EDS analysis

samples	at% Ge	at% Se
bulk glass	24.8 ± 0.2	75.2 ± 0.2
TF 1000 nm	25.66 ± 0.08	74.34 ± 0.08
TF 210 nm	25.37 ± 0.17	74.63 ± 0.17
wires for PAMFT	24.97 ± 0.11	75.03 ± 0.11
samples after PAMFT	24.5 ± 0.9	75.5 ± 0.9

Viscosity measurements

We performed the viscosity measurements in bulk surface and thin films (TF; 1000 nm) using nanoindentation (NI) in glass and undercooled melt regions. To complement the viscosity data in $Ge_{25}Se_{75}$, we also measured viscosities in the melt region using the pressure-assisted melt-filling technique (PAMFT).

In NI measurements, we penetrated the samples using a sapphire Berkovich indenter under a constant applied load (*F*, in a range of 50 – 5000 μN) at a chosen temperature. We select the measurements' applied force and time interval concerning the expected viscosity value. The penetration depth in TF was usually lower than 200 nm to prevent the influence of the substrate. Figure S1a shows the typical measurement. We evaluated the viscosity (η) values from the dependence of penetration depth (h) on time (t):[1, 2]

$$\eta = \frac{F}{4 \cdot \frac{\partial h^2}{\partial t} \cdot \tan\frac{\psi}{2}} \qquad \text{(S1)},$$

where F is the applied load, ψ is the tip angle of the indenter. We took the viscosity value from the steady part of the calculated η vs. t curve (blue data in Figure S1a). Table S2 summarizes the evaluated viscosity data.

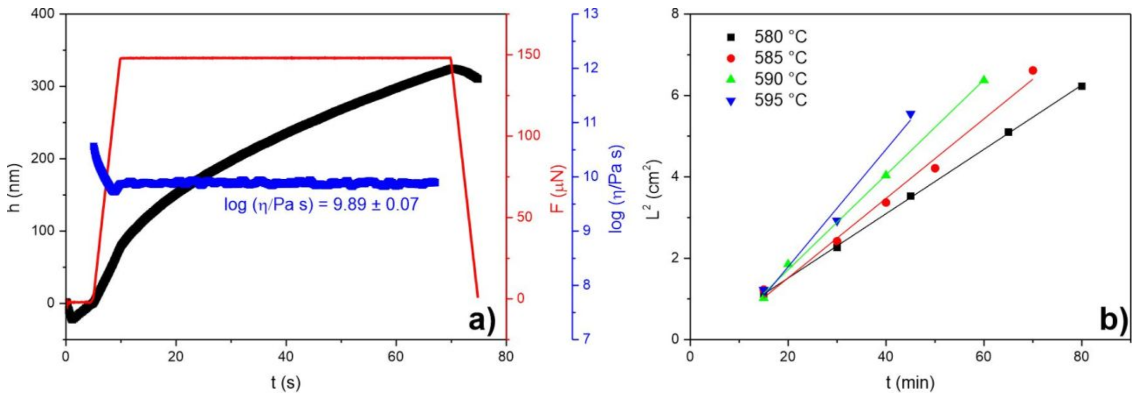

Figure S1 Typical experimental data for viscosity evaluation using a) NI measurements in thin films and bulk surface (data corresponds to bulk at 260 °C), and b) PAMFT measurements in melts at different temperatures (data correspond to 40 bar applied pressure and capillary diameter of 5.8 μm).

We studied the viscous behavior in $Ge_{25}Se_{75}$ melts using the PAMFT described in our previous works.[3-5] PAMFT measurements use the filling of the $Ge_{25}Se_{75}$ melt into a small silica capillary under high Ar pressure. We prepared thin wires (approximately 60 - 80 μm in diameter) of the

$Ge_{25}Se_{75}$ by drawing from melt under the inert atmosphere of N_2. We checked the prepared thin wires, as well as the composition of the material filled into the capillaries after PAMFT measurements using energy dispersive spectrometry (EDS) coupled with a scanning electron microscope (SEM). The composition in all cases corresponded well with the starting $Ge_{25}Se_{75}$ bulk material within the range of 25 ± 1 at% of Ge in Se. We measured the filled capillary length (L) as a function of time (t) at several temperatures. According to our previous works,[4, 5] we chose such a combination of the filled capillary diameter and applied pressure to eliminate the need for wetting properties between the melt and capillary. Then, we calculated the viscosity data from the linear dependence of L^2 on t (Figure S1b) using the simplified formula:[3, 4]

$$L^2 = \left(\frac{p \cdot R^2}{4\eta} \right) \cdot t \quad \text{(S2).}$$

The viscosity data from the PAMFT experiments are listed in Table S2.

Table S2 Viscosity data in $Ge_{25}Se_{75}$ bulk surface and TF measured by NI and in melts measured by PAMFT

PAMFT		NI bulk surface		NI TF	
T [°C]	log (η / Pa s)	T [°C]	log (η / Pa s)	T [°C]	log (η / Pa s)
580	1.89 ± 0.12	200	12.86 ± 0.14	200	12.56 ± 0.08
585	1.86 ± 0.15	205	12.63 ± 0.22	205	12.39 ± 0.09
590	1.70 ± 0.05	210	12.27 ± 0.14	210	12.32 ± 0.05
595	1.53 ± 0.02	215	12.08 ± 0.11	215	12.04 ± 0.05
		220	11.76 ± 0.03	220	11.76 ± 0.07
		225	11.45 ± 0.02	225	11.48 ± 0.11
		230	11.17 ± 0.02	230	11.0 ± 0.4
		235	10.83 ± 0.10	235	10.9 ± 0.4
		240	10.82 ± 0.13	240	10.9 ± 0.4
		245	10.61 ± 0.16	245	10.5 ± 0.4
		250	10.32 ± 0.12	250	10.3 ± 0.3
		255	10.11 ± 0.09	255	10.14 ± 0.11
		260	9.89 ± 0.07		
		265	9.45 ± 0.10		
		270	9.19 ± 0.08		
		275	8.96 ± 0.06		
		280	8.79 ± 0.08		
		285	8.56 ± 0.07		
		290	8.31 ± 0.11		

The viscosity data were fitted by Mauro-Yue-Ellison-Gupta-Allan (MYEGA; Equation 1 in the Manuscript)[6] and Vogel-Fulcher-Tammann (VFT, Equation S1)[7-9] expressed using parameters T_{12} (viscosity glass transition temperature), η_0 (viscosity at infinite temperature) and m (steepness index)[6]:

$$\log \eta = \log \eta_0 + \frac{(12 - \log \eta_0)^2}{m(T/T_{12} - 1) + 12 - \log \eta_0} \quad \text{(S1)}$$

MYEGA and VFT model were used to describe viscous behavior in $Ge_{25}Se_{75}$ material. Because the viscosity data of $Ge_{25}Se_{75}$ bulks and TF corresponded well in the undercooled melt region, we fit all the data together with one model covering the regions of glass, undercooled melt, and melt (Figure S2). The corresponding parameters are listed and compared in Table S3.

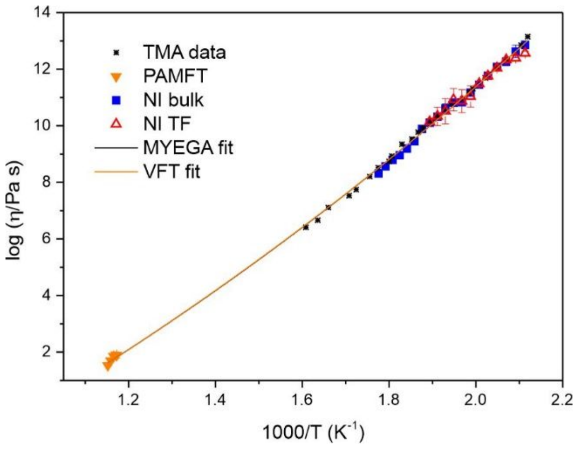

Figure S2 Temperature (T) dependence of near-surface viscosity (η) in $Ge_{25}Se_{75}$ bulks and thin films (TF) obtained by nanoindentation (NI) and melt viscosities obtained by pressure-assisted melt filling technique (PAMFT). The figure shows the comparison with literature data in bulks measured by thermomechanical analysis (TMA) and published by Honcová et al.[10]. All the data were fitted together using MYEGA and VFT models.

Table S3 Parameters of MYEGA and VFT model describing viscous behavior in $Ge_{25}Se_{75}$

	MYEGA	VFT
T_{12} (K)	488.8 ± 0.4	488.8 ± 0.4
m	27.7 ± 0.4	27.8 ± 0.4
log (η_0/Pa s)	-7.6 ± 0.4	-8.1 ± 0.4

R^2	0.99801	0.99798

Crystal growth in $Ge_{25}Se_{75}$ samples

As the main article states, the crystals formed thin plates or some aggregates of thin plates. Therefore, we measured the crystal size (l) as the plate crystals' maximal length or the symmetric aggregates' diameter (d; see the insets in Figure S3). Crystals formed in the amorphous samples grew linearly with time (Figure S3), corresponding to the crystal growth driven by the melt–crystal interphase. The slope of the linear dependence of crystal size (l or d) on time (t) corresponds to the crystal growth rate (u). For in-situ measurements, crystal growth rates were evaluated for several crystals (10-20) found in the sample, and the final growth rate was taken as an average of the evaluated values. In the case of ex-situ measurements, the size of several crystals (10-50) was measured, and their mean value was obtained. The mean value was then plotted as a dependence of annealing time, and the slope gave us the crystal growth rate value. Tables S4 and S5 summarize the crystal growth rates in different forms of $Ge_{25}Se_{75}$ amorphous samples in a wide temperature range of 250 – 560 ˚C.

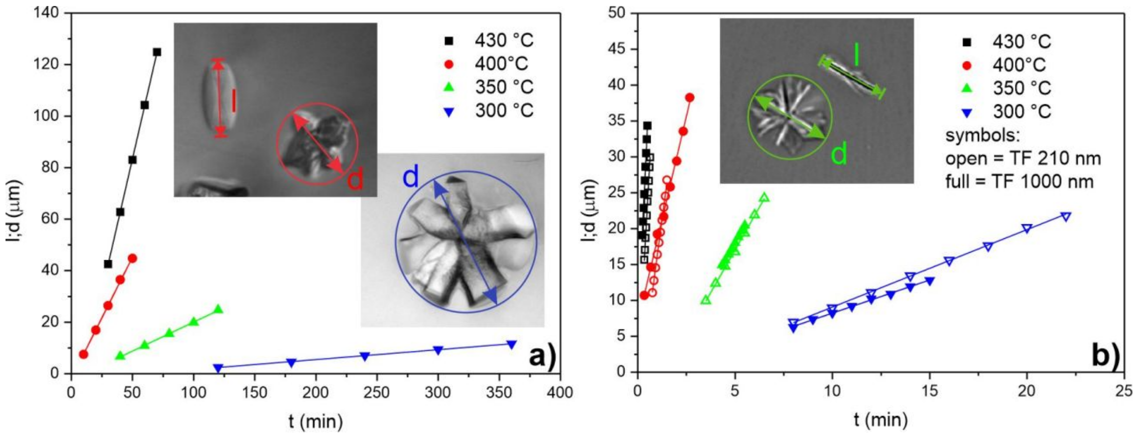

Figure S3 Linear time dependence of crystal size measured in different amorphous samples of $Ge_{25}Se_{75}$ glass-former a) bulk samples, b) thin films of different thicknesses. The insets show typical size measurements of the formed crystals (see the text for more details).

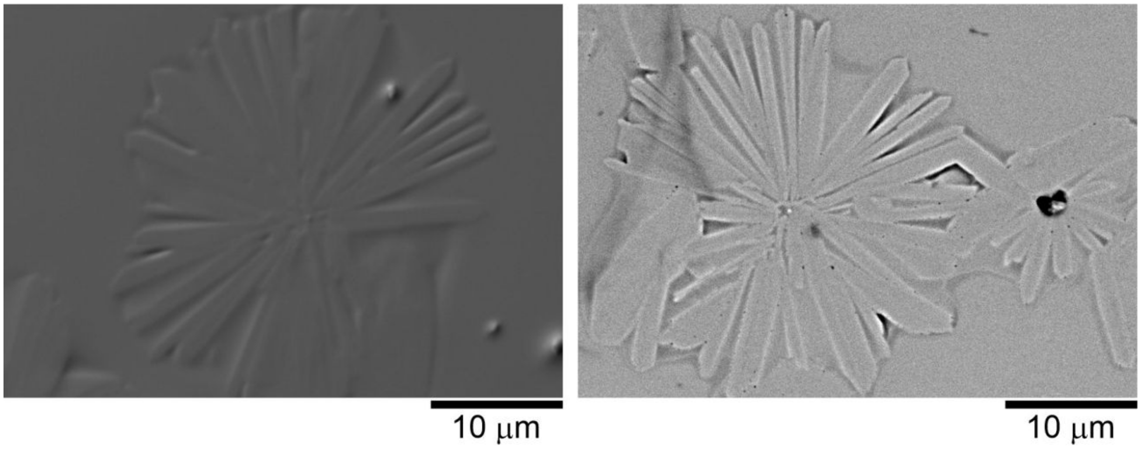

Figure S4 SEM images of crystals grown in TF. Left - topography image showing that the crystal is still covered by a thin amorphous layer. Right – the partially crystallized TF was removed from the surface using tape, and the image was taken from the "bottom" of the TF. Both images were taken using a backscattered electron detector at 10 kV, and no additional coating was used for the measurement.

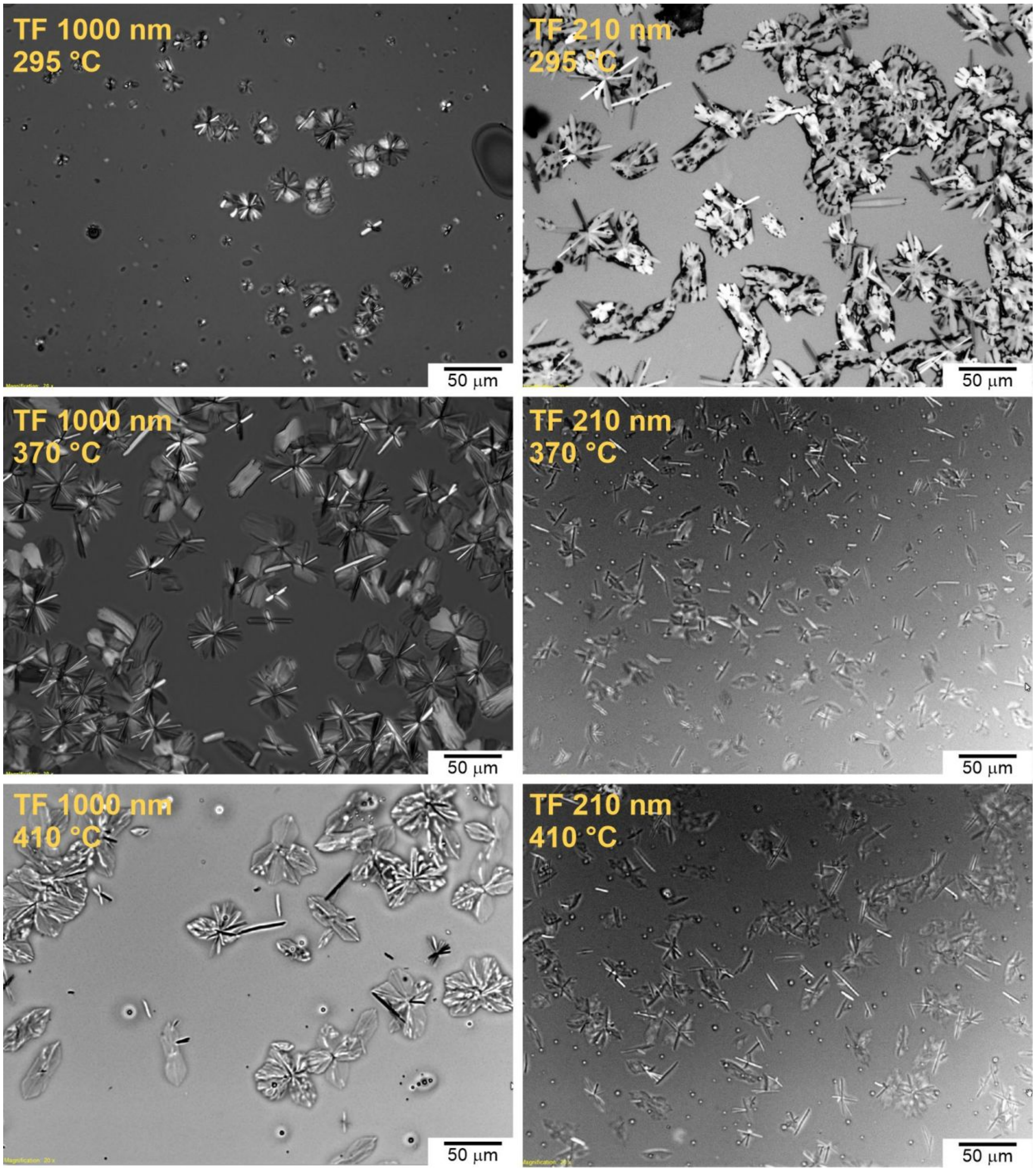

Figure S5 Typical crystals grown in $Ge_{25}Se_{75}$ thin films captured at different temperatures.

Table S4 Crystal growth rates in $Ge_{25}Se_{75}$ bulk samples

T [°C]	u [μm min^{-1}]	T [°C]	u [μm min^{-1}]	T [°C]	u [μm min^{-1}]
295	0.0328 ± 0.0009	415	1.42 ± 0.02	540	12.1 ± 0.2
300	0.0384 ± 0.0005	420	1.608 ± 0.006	542	11.5 ± 0.5
305	0.0454 ± 0.0012	421	1.65 ± 0.019	550	9.9 ± 0.2
310	0.0566 ± 0.0005	425	1.808 ± 0.005	552	8.8 ± 0.4
315	0.0683 ± 0.0013	430	2.061 ± 0.012	559	7.21 ± 0.11
321	0.0826 ± 0.0011	440	2.16 ± 0.04		
325	0.101 ± 0.008	445	2.74 ± 0.15		
332	0.1205 ± 0.0006	449	2.464 ± 0.015		
334	0.1235 ± 0.0006	455	3.21 ± 0.14		
341	0.1622 ± 0.0005	457	3.68 ± 0.09		
344	0.1748 ± 0.0014	466	4.08 ± 0.03		
347	0.169 ± 0.005	468	4 ± 0.07		
348	0.184 ± 0.014	477	5.3 ± 0.2		
352	0.225 ± 0.004	486	6.3 ± 0.2		
353	0.2402 ± 0.0013	489	6.9 ± 0.3		
361	0.31 ± 0.003	495	7.77 ± 0.17		
364	0.326 ± 0.005	500	7.8 ± 0.3		
372	0.417 ± 0.003	505	9.95 ± 0.12		
374	0.447 ± 0.005	509	10.78 ± 0.1		
381	0.561 ± 0.004	515	12.4 ± 0.2		
384	0.602 ± 0.012	519	13.17 ± 0.12		
390	0.73 ± 0.03	523	15.6 ± 0.4		
394	0.8089 ± 0.0004	528	16.46 ± 0.08		
400	0.941 ± 0.015	532	13.87 ± 0.1		
405	1.0878 ± 0.0014	533	9.36 ± 0.05		
411	1.282 ± 0.003	534	12.7 ± 0.4		

Table S5 Crystal growth rates in $Ge_{25}Se_{75}$ thin films (TF)

TF 1000 nm		TF 1000 nm		TF 210 nm	
T [°C]	u [μm min^{-1}]	T [°C]	u [μm min^{-1}]	T [°C]	u [μm min^{-1}]
260	0.13 ± 0.02	395	19.3 ± 0.6	250	0.091 ± 0.004
265	0.184 ± 0.016	400	26.6 ± 1.9	260	0.155 ± 0.003
270	0.2259 ± 0.0018	410	34.1 ± 1.9	270	0.25 ± 0.03
275	0.32 ± 0.05	415	39 ± 4	280	0.383 ± 0.008
280	0.45 ± 0.08	420	44.8 ± 1.1	290	0.569 ± 0.002
285	0.37 ± 0.02	425	58 ± 7	300	0.8 ± 0.04
290	0.55 ± 0.08	430	55 ± 3	310	1.155 ± 0.012
295	0.76 ± 0.03	435	73 ± 4	320	1.61 ± 0.06
300	0.83 ± 0.19	440	76 ± 5	330	2.59 ± 0.14
305	1.22 ± 0.16	445	86 ± 9	340	3.14 ± 0.08
310	1.5 ± 0.2	450	93 ± 3	350	4.68 ± 0.08
315	1.8 ± 0.4	460	122 ± 2	360	6.47 ± 0.06
320	2 ± 0.3	470	150.2 ± 1.5	370	8.4 ± 0.2
325	2.12 ± 0.18	480	193 ± 5	380	11.18 ± 0.08
330	2.6 ± 0.4	490	243 ± 4	390	15.4 ± 0.9
335	3.5 ± 1.3			400	20.2 ± 0.5
340	4.7 ± 1.0			405	23.6 ± 0.3
345	4.5 ± 1.6			410	26.9 ± 1.0
350	5.1 ± 1.3			420	35.1 ± 0.4
355	5.7 ± 1.6			430	45.8 ± 1.4
360	6.1 ± 0.4				
365	7.8 ± 1.6				
370	8.7 ± 0.9				
375	13.2 ± 0.9				
380	11.4 ± 1.5				
385	17.3 ± 1.5				
390	15 ± 4				

Activation energy of crystal growth

Figure S5 shows the linearized Arrhenius model as a dependence of log u on reciprocal temperature (1000/T). The crystal growth rates in TF of 210 nm in thickness provided the same values of crystal growth rates found in TF of 1000 nm. Therefore, we used all the growth data in TF as one data set for further kinetic analysis. The dependence shows a linear character.

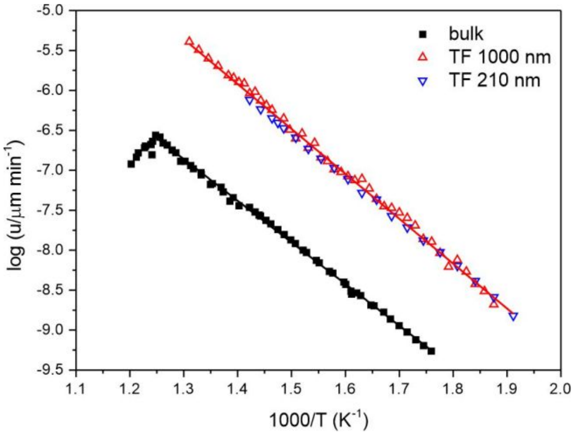

Figure S6 The linearized dependence of crystal growth rate (u) on temsperature (T) in $Ge_{25}Se_{75}$ bulk and TF samples.

References:

(1) Yang, F. Q.; Li, J. C. M. Newtonian viscosity measured by impression test. *J. Non-Cryst. Solids* **1997**, *212* (2-3), 126-135. DOI: 10.1016/S0022-3093(96)00656-4.

(2) Yang, F. Q.; Li, J. C. M. Viscosity of selenium measured by impression test. *J. Non-Cryst. Solids* **1997**, *212* (2-3), 136-142. DOI: 10.1016/S0022-3093(97)00002-1.

(3) Wang, S. Y.; Jain, C.; Wondraczek, L.; Wondraczek, K.; Kobelke, J.; Troles, J.; Caillaud, C.; Schmidt, M. A. Non-Newtonian flow of an ultralow-melting chalcogenide liquid in strongly confined geometry. *Appl. Phys. Lett.* **2015**, *106* (20), 1-5, Article. DOI: 10.1063/1.4921708.

(4) Barták, J.; Košťál, P.; Valdés, D.; Málek, J.; Wieduwilt, T.; Kobelke, J.; Schmidt, M. A. Analysis of viscosity data in As_2Se_3, Se and $Se_{95}Te_5$ chalcogenide melts using the pressure assisted melt filling technique. *J. Non-Cryst. Solids* **2019**, *511*, 100-108, Article. DOI: 10.1016/j.jnoncrysol.2019.01.037.

(5) Košťál, P.; Barták, J.; Wieduwilt, T.; Schmidt, M. A.; Málek, J. Viscosity and fragility of selected glass-forming chalcogenides. *J. Non-Cryst. Solids* **2022**, *575*, 1-11. DOI: 10.1016/j.jnoncrysol.2021.121205.

(6) Mauro, J. C.; Yue, Y. Z.; Ellison, A. J.; Gupta, P. K.; Allan, D. C. Viscosity of glass-forming liquids. *P. Natl. Acad. Sci. USA* **2009**, *106* (47), 19780-19784. DOI: DOI 10.1073/pnas.0911705106.

(7) Vogel, H. Das Temperaturabhängigkeitsgesetz der Viskosität von Flüssigkeiten. *Phys. Z.* **1921**, *22*, 645-646.

(8) Fulcher, G. S. Analysis of recent measurements of the viscosity of glasses. *J. Am. Ceram. Soc.* **1925**, *8* (6), 339-355. DOI: 10.1111/j.1151-2916.1925.tb16731.x.

(9) Tammann, G.; Hesse, W. Die Abhängigkeit der Viscosität von der Temperatur bei unterkühlten Flüssigkeiten. *Z. Anorg. Allg. Chem.* **1926**, *156* (1), 245-257. DOI: 10.1002/zaac.19261560121.

(10) Honcová, P.; Košťál, P.; Včeláková, M.; Svoboda, R.; Sádovská, G.; Barták, J.; Málek, J. Structural interpretation of the viscous flow and relaxation kinetics in the As-Se and Ge-Se chalcogenide systems. *J. Non-Cryst. Solids* **2024**, *643*, 123188. DOI: https://doi.org/10.1016/j.jnoncrysol.2024.123188.
